# GDF15 promotes the proliferation of cervical cancer cells by phosphorylating AKT1 and Erk1/2 through the receptor ErbB2

**DOI:** 10.1186/s13046-018-0744-0

**Published:** 2018-04-10

**Authors:** Shan Li, Yan-Min Ma, Peng-Sheng Zheng, Ping Zhang

**Affiliations:** 1 Department of Reproductive Medicine, the First Affiliated Hospital, College of Medicine, Xi’an Jiaotong University, Shaanxi, Xi’an, 710061 People’s Republic of China; 2 Section of Cancer Stem Cell Research, Key Laboratory of Environment and Genes Related to Diseases, Ministry of Education of the People’s Republic of China, Xi’an, People’s Republic of China

**Keywords:** GDF15, Proliferation, ErbB2, Cervical cancer

## Abstract

**Background:**

Growth differentiation factor 15 (GDF15) is a member of the TGF-β superfamily, and evidence suggests that a substantial amount of GDF15 is secreted in various human cancers, such as ovarian cancer, prostate cancer, and breast cancer, among others. However, the function of GDF15 in cervical cancer has not yet been reported.

**Methods:**

Immunohistochemistry was used to detect GDF15 expression in normal cervix and in different cervical cancer lesions. Cell growth curves, MTT, tumor formation assays and flow cytometry were utilized to observe the effects of ectopic GDF15 expression on the proliferation and cell cycle of cervical cancer cells. Real-time PCR, western blotting and immunoprecipitation assays were conducted to measure the expression of genes related to the cell cycle and the PI3K/AKT and MAPK/ERK signaling pathways. A chromatin immunoprecipitation assay was performed to confirm whether C-myc bound to a specific region of the GDF15 promoter. Inhibitor treatment and immunoprecipitation assays were employed to identify the association between GDF15 and ErbB2.

**Results:**

GDF15 expression gradually increased during the progression of cervical carcinogenesis. GDF15 promoted cervical cancer cell proliferation via exogenous rhGDF15 treatment or the use of gene editing technology in vitro *and* in vivo and significantly accelerated the cell cycle transition from G0/G1 to S phase. The expression of p-ErbB2, p-AKT1, p-Erk1/2, CyclinD1 and CyclinE1 was up-regulated and the expression of p21 was down-regulated in GDF15-overexpressing and rhGDF15-treated cervical cancer cells. C-myc trans-activated GDF15 expression by binding to the E-box motifs in the promoter of GDF15 and contributed to the positive feedback of GDF15/C-myc/GDF15. Furthermore, GDF15 bound to ErbB2 in a protein complex in cervical cancer cells.

**Conclusions:**

Our data demonstrated that GDF15 promoted the proliferation of cervical cancer cells via the up-regulation of CyclinD1 and CyclinE1 and the down-regulation of p21 through both the PI3K/AKT and MAPK/ERK signaling pathways in a complex with ErbB2.

**Electronic supplementary material:**

The online version of this article (10.1186/s13046-018-0744-0) contains supplementary material, which is available to authorized users.

## Background

Currently, cervical cancer is the second most common cancer in women in developing countries after breast cancer, and recent findings indicate an increased risk for young women under 25 years of age who may be predisposed to develop cervical cancer [1]. Multistep factors such as proto-oncogene activation, tumor suppressor gene inactivation, epigenetic alterations, and genomic instability are involved in the development of cervical cancer [2, 3]. Substantial evidence has indicated that various oncogenes (such as *LGR5* and *DAX1*) and suppressor genes (such as *KLF4* and *SLUG*) exhibit abnormal expression during the development and progression of cervical carcinoma [4–7], which suggests the important role of gene activity in the carcinogenesis of cervical cancer. Although it is known that cervical carcinogenesis is a process in which normal tissues progress to squamous intraepithelial lesions and then cervical cancer, the underlying mechanisms of cervical carcinogenesis have not been fully elucidated.

Growth differentiation factor 15 (GDF15, also known as MIC-1, NAG-1, PTGF-β, and PDF) belongs to the transforming growth factor beta (TGF-β) superfamily. The *GDF15* gene is located on chromosome 19 and comprises two exons that encode a 308-amino-acid polypeptide, which consists of a 29-amino-acid signal peptide, a 112-amino-acid mature protein, and a 167-amino-acid pro-peptide. GDF15 is expressed in the cytoplasm as a precursor 35-kDa protein that is cleaved to produce a mature 17-kDa secreted cytokine [8]. GDF15 is weakly and stably expressed in most tissues under normal physiological conditions but is substantially up-regulated under pathological conditions such as injury, inflammation and carcinoma, among other diseases [9]. Evidence shows that GDF15 plays an important role in carcinogenesis-related activities, including proliferation, migration, invasion, and angiogenesis in various types of tumors. For example, GDF15 knockdown in malignant gliomas reduced cell proliferation in vitro and tumorigenesis in vivo [10], while GDF15 overexpression promoted tumorigenesis and progression in oral squamous cell carcinoma [11]. GDF15 was identified as a novel potential biomarker of cervical cancer in a previous study [12]. Unfortunately, there is no evidence demonstrating the effects of GDF15 expression on the development and progression of cervical carcinoma; the molecular mechanisms of GDF15 in cervical carcinoma are also largely unknown. This study aimed to fully explore the function and mechanisms of GDF15 in cervical carcinogenesis.

## Methods

### Cell lines and culture conditions

Five cervical cancer cell lines HeLa, SiHa, C-33 A, HT-3, CaSki and HL-60 were obtained from American Type Culture Collection (ATCC, Rockville, MD, USA) and maintained in recommended media supplemented with 10% fetal bovine serum at 37 °C and 5% CO_2_. Dulbecco’s modified Eagle’s medium (DMEM; Sigma-Aldrich, St. Louis, MO, USA) was used to culture HeLa, SiHa and C-33 A cells, McCoy 5A medium (Sigma-Aldrich) was used for HT-3 cells, RPMI 1640 (Sigma-Aldrich) was used for the CaSki and HL-60 cells under the identical conditions. Recombinant human GDF15 (rhGDF15; 213-10,128-1) and Human serum albumin solution (HSA; 020682) was purchased from RayBiotech (Norcross, GA, USA) and Vitrolife (Gothenburg, Sweden), respectively. MK-2206 is an AKT inhibitor, SCH772984 is an Erk1/2 inhibitor, SB525334 is a TGF-β receptor inhibitor and CI-1033 is an EGFR/ErbB2 inhibitor, all of which were purchased from biochemical company (Selleck, USA).

### Human tissue specimens, immunohistochemistry and immunocytochemistry

A total of 76 samples, 21 normal cervix (NC), 23 high-grade squamous intraepithelial lesion (HSIL), and 32 squamous cervical cancer (SCC) samples without a history of chemotherapy, immunotherapy, or radiotherapy were obtained by means of surgery at the First Affiliated Hospital of Xi’an Jiaotong University Medical College between January 2008 and December 2016. The procedures followed medical-ethics approval practices. Hematoxylin and eosin-stained sections were used for pathological diagnosis. Clinical stages were identified according to the International Federation of Gynecology and Obstetrics (FIGO) classification system. For IHC, the staining procedure was performed as previously described [6]. Images were captured with an Olympus-CX31 microscope digital camera and Leica DFC 500 digital camera and processed with LAS AF software (Leica, Solms, Germany). To determine immuno-reactivity scores (IRSs), specimen sections were scored and evaluated by 2 investigators. Staining intensity was scored on a scale from 0 to 3: negative (0), weak (1), moderate (2), or strong (3). The percentage of positive cells, defined as the relative positive-staining area, was scored on a scale from 0 to 4: 0–5%(0), 6–25%(1), 26–50%(2), 51–75%(3), and 76–100%(4). The IRS was calculated by multiplying the “staining intensity score” by the “percentage of positive cells”. IRSs ≤2 were defined as GDF15-negative, while IRSs between 3 and 12 (3 ≤ IRS ≤ 12) were defined as GDF15-positive.

### ELISA assay

The cell culture media was collected and prepared as 10 times dilution. The detailed procedures were conducted strictly with the protocol in the Quantikine® ELISA human GDF15 immunoassay kit (#DGD150, R&D system, Minneapolis, MN, USA).

### Western blotting analysis

Western blotting analyses were performed as previously described using 30μg lysates from fresh tissues and cells [6]. Proteins were visualized by exposing Chemiluminescent HRP Substrate (Millipore, Billerica, MA, USA) by protein imprinting imaging system (Tanon5200, China). Antibodies against human GDF15, AKT1, Erk1/2, GAPDH were purchased from Santa Cruz Biotechnology (Dallas, TX, USA). Anti-PI3K (p-p110a) was from Abcam Technology (Cambridge, MA, USA). Anti-p-AKT was purchased from CST (Littleton, CO, USA). Anti-Ras and Anti-Ras-GTP was from NewEast Biosciences (WuHan, CN). Anti-p-Erk1/2, anti-ErbB2, anti-p-ErbB2, anti-FOXO1 were from Abclonal Technology (Boston, MA, USA).

### Plasmid construction and stable transfectants

The vector pIRES2-AcGFP was purchased from Clontech (CA, USA). The full-length CDS of human GDF15 was cloned into the pIRES2-AcGFP vector. GDF15-specific and ErbB2-specific short hairpin RNAs were designed and cloned into pGPU6/GFP vectors to inhibit GDF-15 and ErbB2 expression (Gene Pharmcompany, Shanghai, China). The primers and oligonucleotide sequences are described in Supplementary Additional file 1: Table S1. The pX330-U6-Chimeric_BB-CBh-hSpCas9 plasmid (Plasmid#42230) containing SpCas9 and single guide RNA was obtained from a non-profit plasmid share repository belonging to Feng Zhang (Addgene, Cambridge, MA, USA) [13]. Suitable CRISPR target sites within the positive and negative strands of exon 1 of GDF15 were identified using the ‘CRISPR Design Tool’ (http://crispr.mit.edu/) hosted by the Feng Zhang laboratory (Massachusetts Institute of Technology, MA, USA) to obtain the minimum number of off-target sites in the human genome (Additional file 1: Table S1). To generate stable transfected cell lines, cells were transfected with vectors using Lipofectamine 2000 (Invitrogen, Carlsbad, CA, USA). Stable clones from HeLa and SiHa cells were selected using G418 reagent (Calbiochem, Darmstadt, Germany) and identified by western blotting. For transient HT-3 cell lines, protein was collected after being transfected 48 h.

### RNA isolation and real-time PCR

Total RNA was isolated using TRIzol Reagent (Invitrogen, Carlsbad, CA, USA), and first-strand cDNA was synthesized using the PrimeScript RT Reagent Kit (TaKaRa, Osaka, Japan). Complementary DNA and SYBR-Green fluorescence- signal-detection assays (TaKaRa, Osaka, Japan) were used for real-time PCR. The resulting data were normalized to housekeeping gene GAPDH. Real-time PCR and data collection were performed on an IQ5 instrument (Bio-Rad, CA, USA). Primers were designed using Perl Primer software, and their oligonucleotide sequences are described in Additional file 2: Table S2. The real time-PCR experiments were quantified by the 2 − △△CT method and repeated at least three times.

### Cell growth and cell viability assays

Cells (5 × 10^4^) were seeded in triplicate with 2 mL of media into 35-mm tissue culture dishes for 7 days. The numbers of cells were counted after harvesting every 2 days using a hemocytometer under light microscopy. The cell viability assays were performed by applying 3-(4,5-dimethylthiazole-yl)-2,5-diphenyl tetrazolium bromide (Sigma-Aldrich, St Louis, MO, USA) dye to cells that were seeded in 96-well plates for 1000 cells each in 7 days or 3000 cells in 48 h, as described in a standard protocol. Then, the absorbance at 490 nm was measured (Bio-Rad, CA, USA).

### Flow cytometry analysis

Cells for cell cycle analysis were harvested and fixed with 70% cold ethanol at 4 °C overnight. After being washed in PBS, the cells were incubated in 1 mL of staining solution (20 mg/mL propidium iodide; 10 U/mL RNaseA) (Aldrich, St. Louis, MO, USA) at room temperature for 30 min. Then, the samples were measured by FACS Calibur flow cytometry (BD, Franklin Lakes, NJ, USA), and the cell cycle distributions were analyzed by the software “Flowjo_V10” (FlowJo, LLC, Ashland, OR, USA).

### Quantitative chromatin immunoprecipitation

HeLa and SiHa cells were subjected to ChIP using the EZ-ChIP Assay kit (Millipore, MA, USA). Briefly, the cells were treated with 37% formaldehyde to crosslink proteins, and the reaction was terminated with 0.125 M glycine. After sonication, chromatin–protein complexes were immunoprecipitated with 5 μg of anti-C-myc antibodies or 1 μg of mouse IgG. Real-time PCR was performed to amplify the regions of interest or internal negative control regions. Each sample was assayed in triplicate, and the fold enrichment ratio was calculated as the value of the ChIP sample versus the corresponding input sample. Samples that yielded a twofold enrichment or better were considered positive targets. The primers used for these studies are listed in Additional file 2: Table S2.

### Immunoprecipitation assay

Primary antibody (anti-GDF15) was incubated with AminoLink plus coupling resin (Pierce Co-IP kit, Rockford USA) for 1 h at room temperature. Equivalent amounts of protein (500 μg) incubated with resin-antibody complex overnight at 4 °C. Protein–antibody complexes were washed in lysis buffer. Denatured protein complexes were separated by western blotting. Another antibody(anti-p-ErbB2) was used to detect interacting proteins. IgG antibodies of corresponding isotype (Santa Cruz, Dallas, TX, USA) were used as controls to exclude unspecific associations.

### Tumor xenograft assay

For in vivo assays, nude female mice between 4 and 6 weeks old were purchased from an animal breeding facility (Slac Laboratory Animal Co., Nanjing, CN). Animals were maintained in a specific pathogen free(SPF) room in accord with institutional policies. The mice were randomly divided into four groups, with three repeats for each clone: HeLa-GFP and HeLa-GDF15, SiHa-GFP and SiHa-GDF15. Each mouse received a subcutaneous injection of 1 × 10^6^ cells. The development and progression of solid tumors was monitored longitudinally over 12 weeks. Tumor volume(V) was calculated every 4 days by tumor length(L) and width(W) as: V = L × W^2^/2.

### Statistical analyses

Each sample was assayed in triplicate, and each experiment was repeated three times. Data are presented as means ± SD, and Student’s t-test (unpaired, two- tailed) or one-way ANOVA was used to compare the means of independent samples. Correlations were analyzed using Pearson linear-regression analysis. Statistical analyses were performed with SPSS for Windows v. 16.0 (SPSS Inc., Chicago, IL, USA). *P*-values of *P* < 0.05 were considered statistically significant.

## Results

### GDF15 expression in normal cervix and different cervical cancer lesions

**Fig. 1:**
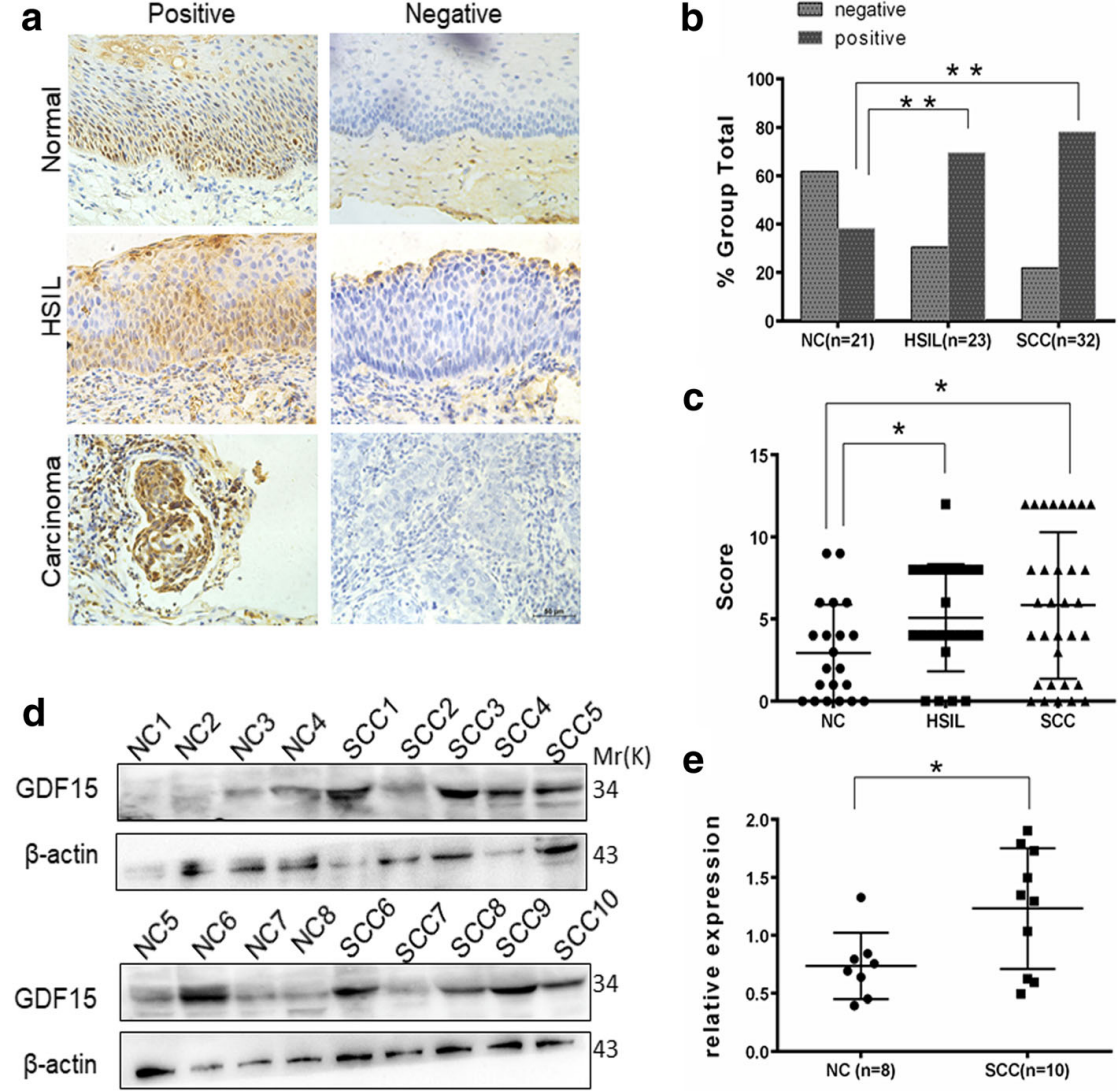
Expression of GDF15 in samples of normal cervix and various cervical lesions. Immuno-histochemical (IHC) detection of GDF15 in normal cervix(NC), high-grade squamous intraepithelial lesion(HSIL) and squamous cervical cancer(SCC) samples (**a**); original magnification, 400×. GDF15 staining was classified into 2 categories, negative and positive (**b**), and the bar chart shows the percentage of each group (21 NC specimens, 23 HSIL specimens, and 32 SCC specimens). The scatter plot illustrates the immunoreactivity scores obtained for GDF15 staining in NC, HSIL and SCC samples (**c**), points represent the IHC score per specimen, and one-way ANOVA was performed. The expression of GDF15 in normal cervix (NC) and squamous cervical carcinoma (SCC) samples was detected by western blotting (**d**). The relative expression of GDF15 in each sample of NC (*n* = 8) and SCC (*n* = 10) is shown (**e**). The data are shown as the ratios of GDF15/β-actin of each specimen and as the means ± standard error of the NC and SCC groups. Values are shown as the mean ± SD, *p < 0.05, **p < 0.01

Although GDF15 expression has been discovered in various carcinomas, its role in cervical cancer is not well defined. To investigate GDF15 protein expression in human cervical carcinoma, immunohistochemistry (IHC) was performed in paraffin -embedded normal cervix (NC), high-grade squamous intraepithelial lesion (HSIL) and squamous cervical cancer (SCC) tissues. Positive GDF15 staining that was localized to the cytoplasm and/or nucleus (Fig. 1a) was found in 38.1% (8/21) of NC samples, in 69.6% (16/23) of HSIL samples and in 78.1% (25/32) of SCC samples (Fig. 1b, NC vs. HSIL, *P* < 0.01; NC vs. SCC, *P* < 0.01; HSIL vs. SCC, *P* > 0.05). The average IHC scores for GDF15 were 2.95 ± 0.64 in NC, 5.09 ± 0.68 in HSIL and 5.84 ± 0.79 in SCC (Fig. 1c, HSIL vs. NC, *P* < 0.05; SCC vs. NC, *P* < 0.05 HSIL vs. SCC, P > 0.05). In a follow up study performed with the TCGA database (TCGA_CESC_exp_HiSeqV2), women with high GDF15 expression showed a statistically significant higher risk of cancer development and shorter disease-free survival time within 10 years compared to women with low GDF15 expression. These data suggested that GDF15 is involved in the development and progression of cervical carcinoma. In addition, the expression of GDF15 in 8 samples of normal cervix and 10 samples of cervical carcinoma was detected by western blotting (Fig. 1d). The relative expression levels of GDF15 in these cervical carcinoma tissues were higher than that in the normal cervix (Fig. 1e, *P* < 0.05). These results all suggested that GDF15 was highly expressed in the examined cervical cancer specimens and may be involved in the process of cervical carcinoma.

### GDF15 promoted the proliferation of cervical cancer cells and tumor formation in vitro and in vivo

**Fig. 2:**
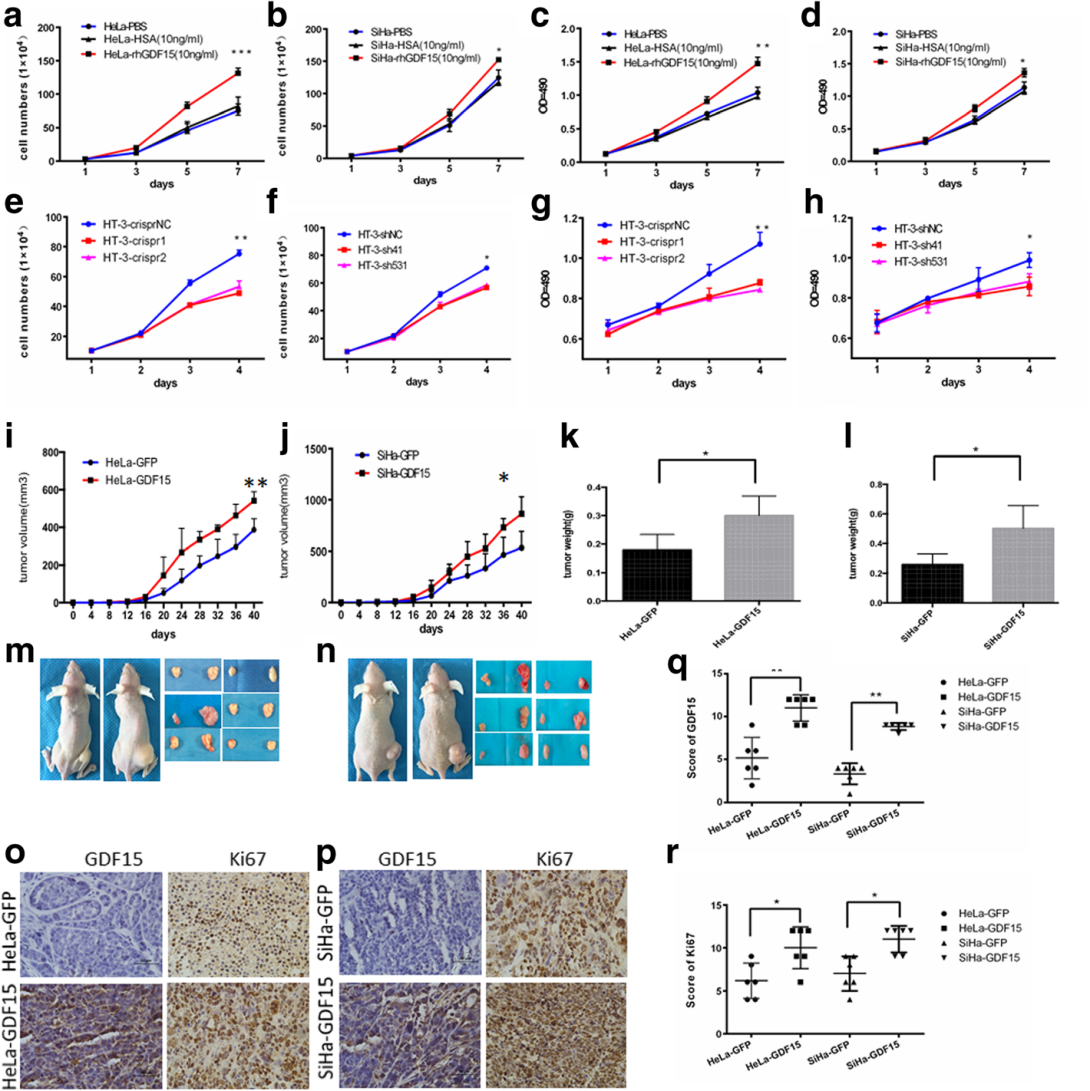
GDF15 promotes cervical cancer cell proliferation in vitro and tumor formation in vivo. The proliferation and viability of HeLa and SiHa cells incubated with PBS, HSA(10 ng/ml) or rhGDF15(10 ng/ml) were detected by growth curves (**a** and **b**, respectively) and the MTT assay (**c** and **d**, respectively). The proliferation and viability of HT-3 cells modified with CRISPR and shRNA were detected by growth curves (**e** and **f**, respectively) and the MTT assay (**g** and **h**, respectively). Tumor growth curves (**i** and **j**, respectively) and tumor weights (**k** and **l**, respectively) are shown for HeLa-GDF15 and SiHa-GDF15 cells and control cells (HeLa-GFP and SiHa-GFP). Pictures were of nude mouse with tumors from HeLa-GDF15 and SiHa-GDF15 and their control cells (**m** and **n**, respectively). Immuno-histochemical staining results for Ki67 are shown in tumor xenografts of GDF15-overexpressing HeLa and SiHa cells **(o** and **p**, respectively) and the quantitative analysis are shown (**q** and **r**, respectively). The data were shown as the mean ± SD of three independent experiments. *p < 0.05, **p < 0.01 vs. control using One-Way ANOVA

A relatively high level of GDF15 expression was detected in HT-3 cells, but relatively low expression of GDF15 was observed in HeLa, SiHa, C-33 A and CaSki cells (Additional file 3: Figure S1a and b) according to IHC and western blotting. To further investigate the function of GDF15 in human cervical cancer cells, commercial recombinant human GDF15 (rhGDF15) and human serum albumin (HSA) was added to the media of HeLa and SiHa. An MTT assay revealed that rhGDF15 stimulated the proliferation of HeLa and SiHa cells in a dose-dependent manner (Additional file 3: Figure S1c and d). Additionally, cell growth curves and an MTT assay were applied to evaluate the proliferation and viability of the HeLa and SiHa cell lines, which were incubated with PBS, HSA or rhGDF15. As shown in Fig. 2a and b, HeLa and SiHa cells treated with rhGDF15 grew more rapidly than their respective controls (HeLa PBS and SiHa PBS, HeLa-HSA and SiHa-HSA, *P* < 0.05). Moreover, the viability of HeLa and SiHa cells treated with rhGDF15 was much higher than that of their respective controls (Fig. 2c and d, P < 0.05). Furthermore, the supernatants of the GFP and GDF15-transfected HeLa and SiHa cell clones (GDF15 concentration in the supernatant of HeLa-GFP and SiHa-GFP is 3.64 ± 0.12 ng/ml and 0.98 ± 0.07 ng/ml; GDF15 concentration in the supernatant of HeLa-GDF15 and SiHa-GDF15 is 38.7 ± 0.83 ng/ml and 19.4 ± 0.51 ng/ml respectively, determined by ELISA assay) were collected and incubated with HeLa-GFP and SiHa-GFP cells for 24 h, 48 h, 72 h and 96 h. An MTT study showed that 50% conditional medium significantly increased the proliferation of HeLa-GFP and SiHa-GFP cells after treatment for 96 h (Additional file 3: Figure S1e and f, *P* < 0.01). Conversely, GDF15 knockdown in HT-3 cells using both CRISPR-CAS9 mediated gene editing and shRNA technologies resulted in significant decreases in cell growth and viability (Fig. 2e to h, *P* < 0.05). All of these results demonstrated that the GDF15 protein stimulated the proliferation of cervical cancer cells in vitro in both an autocrine and a paracrine manner.

**Fig. 3:**
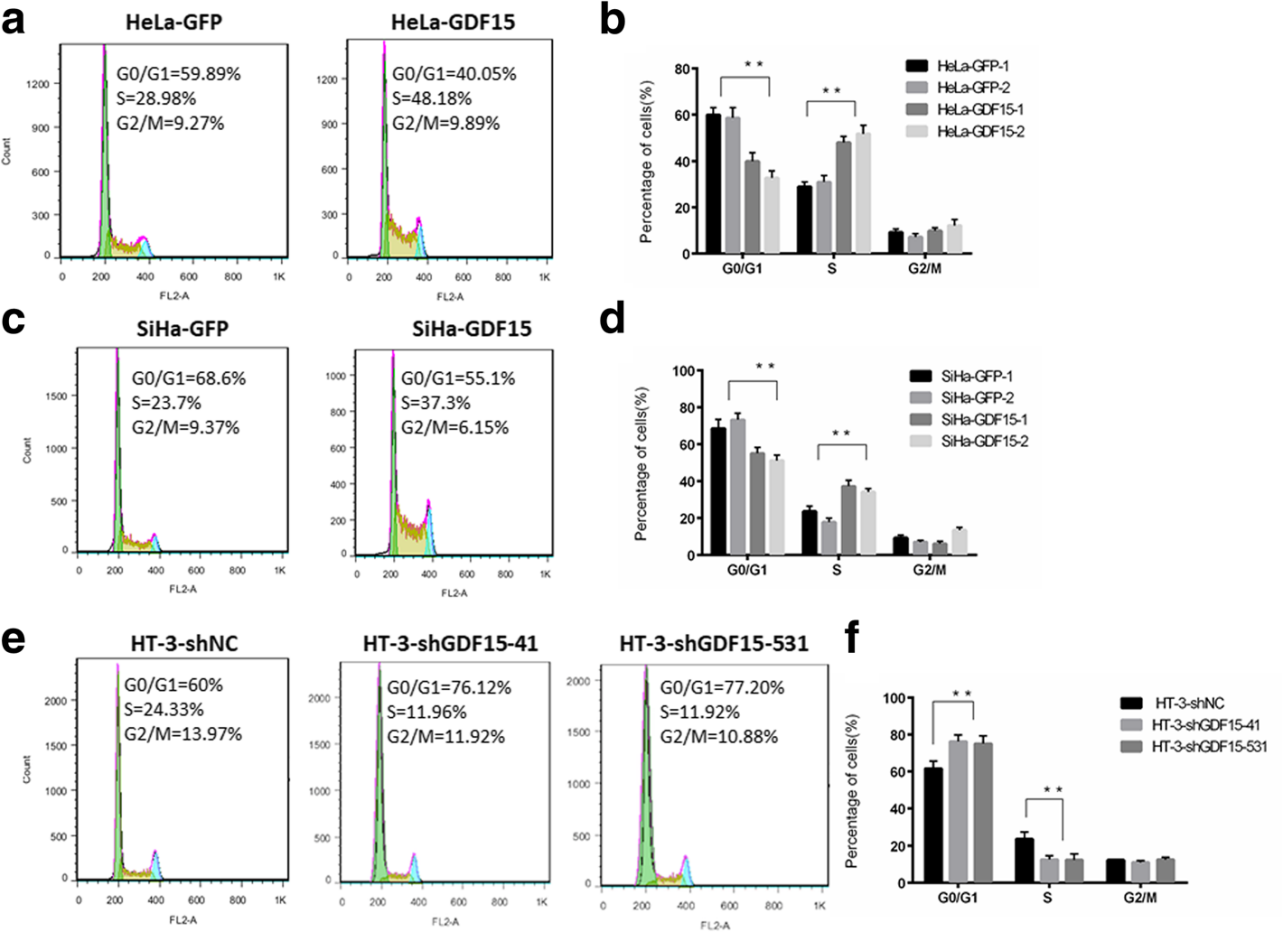
GDF15 accelerated cell cycle transition from G1 to S phase in cervical cancer cells. The cell cycle was analyzed using flow cytometry. The representative cell cycles of HeLa-GDF15 cells (**a**) SiHa-GDF15 cells (**c**) HT-3-shGDF15 (**e**) with their respective controls and the quantitative analysis were shown (**b**, **d** and **f**). The data were shown as the mean ± SD of three independent experiments. *p < 0.05, **p < 0.01 vs. control using One-Way ANOVA

To further determine the effects of GDF15 on tumor formation ability in vivo, 6 adult female nude mice in each group were subcutaneously transplanted with HeLa-GDF15 or SiHa-GDF15 and their control cells (Fig. 2m and n). The xenograft assay in BALB/c-nude mice showed that GDF15-overexpressing cells formed much larger tumors in terms of volume (Fig. 3i and j, *P* < 0.05) and weight (Fig. 3k and l, *P* < 0.05) than did control cells (HeLa-GFP and SiHa-GFP). Furthermore, IHC revealed that the xenograft tumor tissues formed by HeLa-GDF15 and SiHa-GDF15 cells demonstrated much stronger Ki67 staining score than those formed by control cells (HeLa-GFP and SiHa-GFP) (Fig. 3o-r, *P* < 0.05). All of these data suggested that GDF15 promoted tumor formation of cervical cancer cells, potentially via enhanced cell proliferation in vivo.

### GDF15 promoted the proliferation of cervical cancer cells by accelerating the cell cycle transition from G0/G1 to S phase

Generally, the changes that occur during cell proliferation involve modulation of the cell cycle. To investigate whether GDF15 affected the cell cycle of cervical cancer cells, flow cytometry was performed to analyze differences in the cell cycle between GDF15-modified cervical cells and their control cells. As shown in Fig. 3a and b, the percentage of cells in G0/G1 phase obviously decreased from 59.29% in HeLa-GFP cells to 36.43% in HeLa-GDF15 cells, while the percentage of cells in S phase was markedly increased from 30.51% in HeLa-GFP cells to 50.06% in HeLa-GDF15 cells. Consistently, a similar result was observed in SiHa cells; the percentage of cells in G0/G1 phase decreased from 70.95% in SiHa-GFP cells to 53.20% in SiHa-GDF15 cells, and the percentage of cells in S phase increased from 20.80% in SiHa-GFP cells to 35.80% in SiHa-GDF15 cells (Fig. 3c and d, *P* < 0.01). These results suggested that the overexpression of GDF15 accelerated the cell cycle transition from G0/G1 to S phase in cervical cancer cells. Conversely, the percentage of G0/G1 cells significantly increased and the percentage of S phase cells significantly decreased in HT-3-shGDF15 cells compared with HT-3-shNC cells (Fig. 3e and f, P < 0.01), which suggested that GDF15 silencing delayed the cell cycle transition from G0/G1 to S phase. Together, these results showed that GDF15 accelerated the cell cycle in cervical cancer cells at the transition from G0/G1 to S phase and suggested that GDF15 promoted cervical cancer cell proliferation through alterations in the cell cycle phases.

### GDF15 altered the expression of cell cycle-related genes via FOXO1 and C-myc

**Fig. 4:**
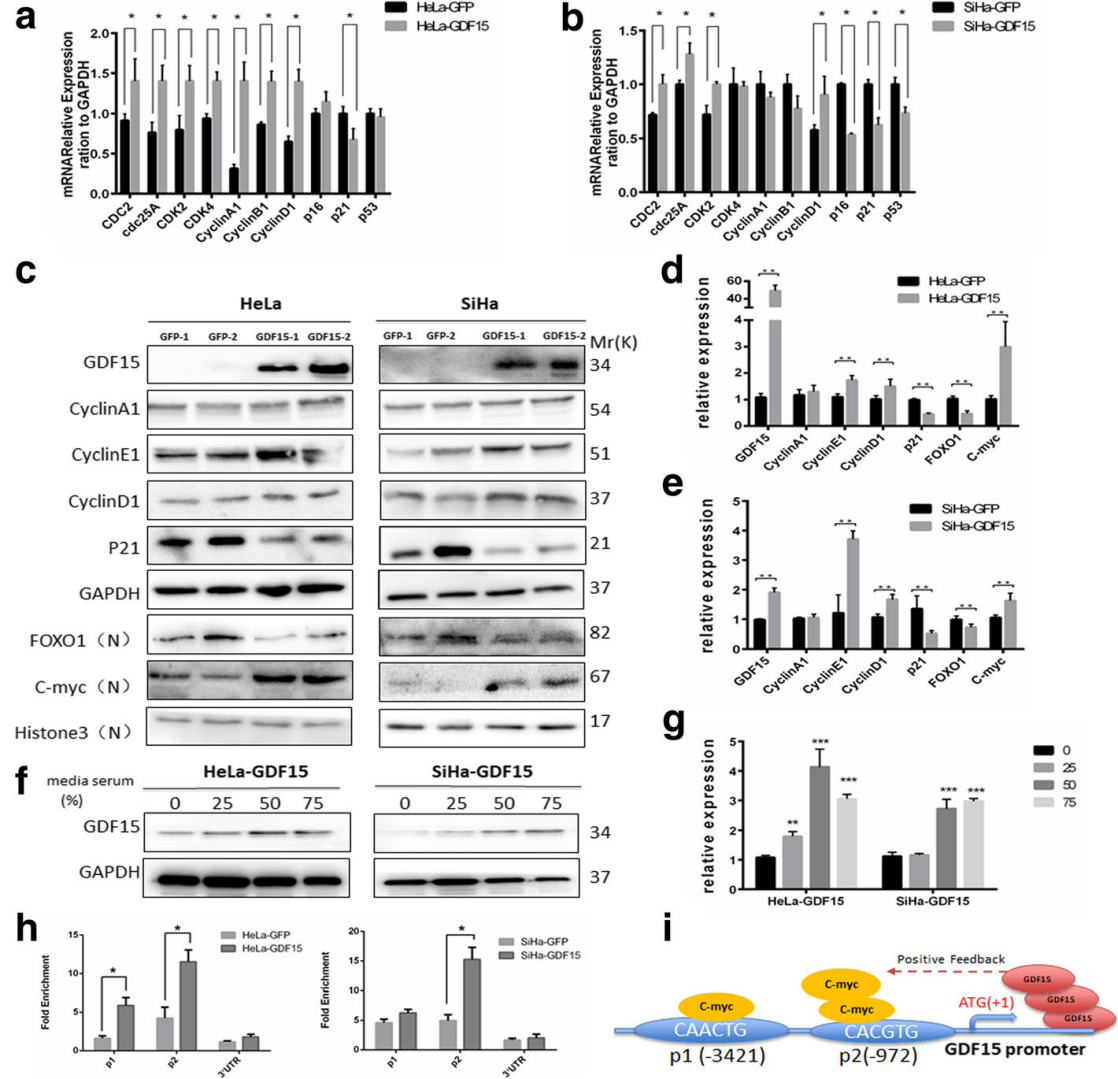
GDF15 altered cell cycle related molecules through FOXO1 and C-myc. The mRNA levels of cell cycle-related genes in HeLa (**a**) and SiHa (**b**) cell lines was detected by quantitative real time-PCR. The total protein levels of GDF15, CyclinA1, CyclinE1, CyclinD1 and p21 and the nuclear protein FOXO1 and C-myc in HeLa and SiHa cell lines (**c**) were determined by western blotting and the quantitative analysis were presented (**d** and **e**). GDF15 was determined by western blotting and cell lysates from HeLa-GDF15 and SiHa-GDF15 cells was incubated with 0%, 25%,50% and 75% conditioned medium for 24 h (**f**) and the quantitative analysis are shown (**g**). A quantitative CHIP assay of the GDF15 promoter region in HeLa and SiHa cells is shown (**h**). Proposed model of the positive feedback relationship between GDF15 and C-myc in cervical cancer cells (**i**). The data were shown as the mean ± SD of three independent experiments. *p < 0.05, **p < 0.01 vs. control using One-Way ANOVA

To explore the detailed mechanisms underlying the effects of GDF15 on the cell cycle, real-time PCR and western blotting were applied to verify the key cell cycle regulators in GDF15-overexpressing cells (HeLa-GDF15, SiHa-GDF15) and their control cells (HeLa-GFP and SiHa-GFP). As shown in Fig. 4a and b, cdc2, cdc25A, CDK2, CDK4 and CyclinD1 mRNA was expressed at much higher levels in HeLa-GDF15 and SiHa-GDF15 cells than in their control counterparts, respectively. Various pathological and HPV types might result in certain differences in CDK4 mRNA expression levels between the two cell lines. In addition, the CyclinD1 and CyclinE1 proteins were also expressed at much higher levels in both HeLa-GDF15 and SiHa-GDF15 cells than in their control counterparts (Fig. 4c-e, *P* < 0.05). Conversely, the expression of p21 was significantly decreased at both the mRNA and protein levels in both HeLa-GDF15 and SiHa-GDF15 cells compared with their respective controls (Fig. 4a-e, P < 0.05).

FOXO1 [14] and C-myc [15, 16] are two well-known classical transcription factors that regulate the cell cycle via their target genes, which include p21, CyclinD1, and CyclinE1, among others. Figure 4c to e shows the decreased expression of FOXO1 and the increased expression of C-myc in both HeLa-GDF15 and SiHa-GDF15 cells, compared with their respective controls (HeLa-GFP and SiHa-GFP). These results strongly suggested that GDF15 accelerated cell cycle progression in cervical cancer cells by targeting cdc25A, CDK2, CDK4, p21, CyclinD1 and CyclinE1 through alterations in the expression of FOXO1 and C-myc. Interestingly, pro-GDF15 expression was further elevated after treatment with conditioned medium from GDF15 overexpressing cells (HeLa-GDF15 and SiHa-GDF15, Fig. 4f and g) and treatment with rhGDF15 (Additional file 4: Figure S2a-d). We speculated that there was mechanism regulating the positive feedback of GDF15 expression. Two transcriptional binding elements (CAACTG at − 3421 and CACGTG at − 972) were detected in the promoter of GDF15 through sequence analysis in the UCSC Genome online database; additionally, the E-box motif (CANNTG) is regulated by the typical transcription factor C-myc. To confirm our suspicion, a quantitative ChIP (chromatin immunoprecipitation) assay was applied to demonstrate that C-myc recognized and bound the promoter to trans-activate the expression of GDF15 (Fig. 4h, *p* < 0.05). The positive feedback between GDF15 and C-myc is shown in Fig. 4i.

### GDF15 induced the activation of AKT and ERK1/2 in human cervical cancer cells

**Fig. 5:**
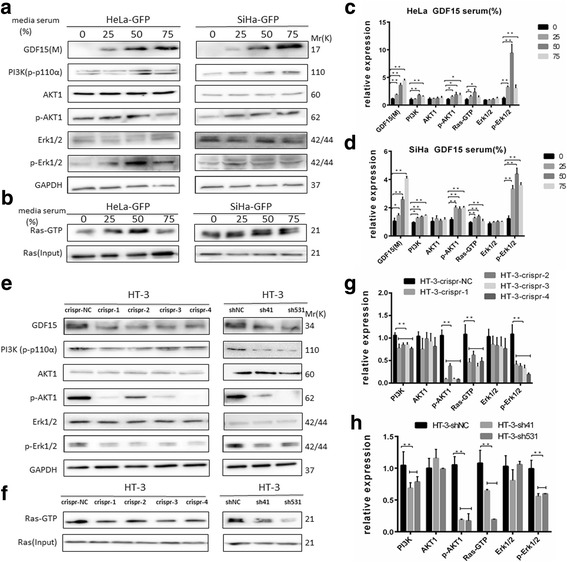
GDF15 induced activation of AKT1 and ERK1/2 in human cervical cancer cell lines. GDF15, PI3K, AKT/p-AKT1, and Erk1/2/p-Erk1/2 were determined by western blotting and Ras-GTP was by immunoprecipitation. Western blotting of cell lysates from HeLa-GFP and SiHa-GFP cells which were incubated with 0%, 25%, 50% and 75% conditioned medium for 24 h (**a** and **b**) and the quantitative analysis are shown (**c** and **d**). Western blotting of cell lysates from HT-3-crispr-GDF-15 and HT-3-shGDF15 and their control cell lines (**e** and **f**) and the quantitative analysis are shown (**g** and **h**). N: nuclear protein; M: medium protein. The data were shown as the mean ± SD of three independent experiments. *p < 0.05, **p < 0.01 vs. control using One-Way ANOVA

It has been demonstrated that the expression of FOXO1 and C-myc is regulated by AKT1 and Erk1/2 through the PI3K/AKT and MAPK/ERK signaling pathways in various human carcinomas [17, 18]. To our knowledge, it has not been determined whether GDF15 promotes the proliferation of cervical cancer cells through the mechanism discussed above. Therefore, in the present study, the expression levels of PI3K (p-p110a), AKT1, p-AKT1 (S473), Erk1/2, p-Erk1/2 (T202/Y204) and Ras-GTP (total active Ras-GTP included H-Ras, N-Ras and K-Ras) were detected by western blotting or immunoprecipitation assay. As shown in Fig. 5a to d, treatment with conditional medium significantly increased the expression of PI3K, p-AKT1, p-Erk1/2 and Ras-GTP in both HeLa and SiHa cells. Consistently, the expression of PI3K, p-AKT1, p-Erk1/2 and Ras-GTP significantly increased as a result of rhGDF15 treatment (Additional file 4: Figure S2a-d *P* < 0.01). Similarly, the expression of PI3K, p-AKT1, p-Erk1/2 and Ras-GTP was much higher in HeLa-GDF15 and SiHa-GDF15 cells than in HeLa-GFP and SiHa-GFP cells (Additional file 4: Figure S2e-h, P < 0.01). Furthermore, GDF15 knockdown in HT-3 cells by CRISPR technology (HT-3-crispr-GDF15) and by shRNA (HT-3-shGDF15) obviously decreased the expression of PI3K, p-AKT1, p-Erk1/2 and Ras-GTP compared with HT-3-crispr-NC and HT-3-shNC cells (Fig. 5e-h, *P* < 0.01). All of these data suggested that GDF15 promoted cervical cancer cell proliferation and tumor formation via activation of the PI3K/AKT and MAPK/ERK signaling pathways.

### GDF15 activated the PI3K/AKT and MAPK/ERK signaling pathways via ErbB2 phosphorylation

**Fig. 6:**
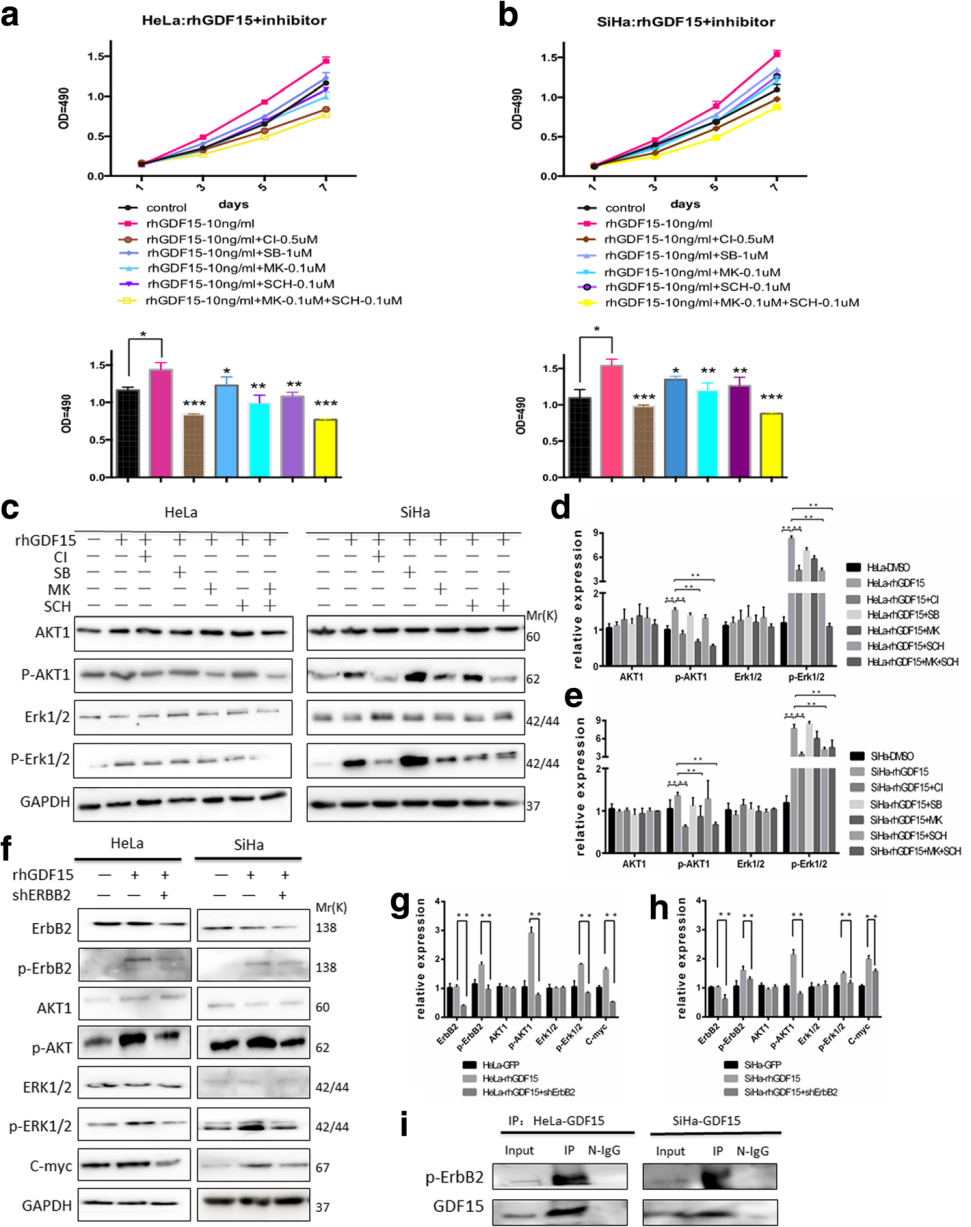
GDF15 stimulated tyrosine phosphorylation of ErbB2. The effects of various inhibitors on HeLa (**a**) and SiHa (**b**) cell proliferation and viability was detected by MTT assay and quantitative statistics in the 7th day were present below, respectively. Western blotting of cell lysates from HeLa and SiHa cells which were treated with various factors (**c**) and the quantitative analysis were shown (**d** and **e**). Western blotting of cell lysates from HeLa and SiHa which were treated with rhGDF15 or/and shErbB2 (**f**) and the quantitative analysis were shown (**g** and **h**). GDF15 was immunoprecipitated from cell lysates of GDF15 overexpressing cell lines (HeLa-GDF15 and SiHa-GDF15) and the immunoprecipitation product was immunoblotted with an anti-p-ErbB2 antibody (**i**). The data were shown as the mean ± SD of three independent experiments. *p < 0.05, **p < 0.01 vs. control using One-Way ANOVA

It has been shown that ErbB2 is transactivated by GDF15 in human breast and gastric cancer cells [19]. ErbB2, a member of the epithelial growth factor receptor (EGFR) family, activates the PI3K/AKT and MAPK/ERK pathways to regulate cell proliferation, migration, and differentiation [20–22]. Moreover, as a member of the TGF-β superfamily, GDF15 regulates cellular biological processes by directly binding to the TGF-β receptor [23]. In the present study, an ErbB2 inhibitor (CI-1033), a TGF-β receptor inhibitor (SB525334), an AKT inhibitor (MK-2206) and an Erk1/2 inhibitor (SCH772984) were applied to investigate the detailed mechanism involving GDF15 in the PI3K/AKT and MAPK/ERK signaling pathways. As shown in Fig. 6a and b, an MTT assay demonstrated that HeLa and SiHa cell proliferation was significantly promoted by rhGDF15 administration but was inhibited by treatment with an AKT inhibitor or an Erk1/2 inhibitor. Notably, the combination of the AKT inhibitor and the Erk1/2 inhibitor induced more potent inhibition of HeLa and SiHa cell proliferation. Additionally, both the TGF-βR inhibitor and the ErbB2 inhibitor significantly suppressed the proliferation of HeLa and SiHa cells, but the ErbB2 inhibitor had a more potent inhibitory role than the TGF-βR inhibitor. Furthermore, the western blotting results showed that the expression of p-AKT1 and p-Erk1/2 was decreased by ErbB2 inhibitor treatment but not by TGF-βR inhibitor treatment in HeLa and SiHa cells (Fig. 6c-e, *P* < 0.01). In addition, the expression of p-AKT1, p-Erk1/2 and C-myc was much lower in HeLa-shErbB2 and SiHa-shErbB2 cells than in HeLa-shNC and SiHa-shNC cells (Fig. 6f-h, *P* < 0.01). Consistently, ErbB2 inhibitor treatment significantly reduced the expression of p-ErbB2 (Y1221/1222), p-AKT1, p-Erk1/2 and C-myc in HeLa and SiHa cells (Additional file 5: Figure S3, *P* < 0.01). Furthermore, an immunoprecipitation assay showed that GDF15 bound to p-ErbB2 in both HeLa-GDF15 and SiHa-GDF15 cells (Fig. 6i). Together, these results suggested that GDF15 activated the PI3K/AKT and MAPK/ERK signaling pathways through ErbB2 in cervical cancer cells.

### Correlation analysis between the expression of GDF15 and p-AKT1, p-Erk1/2, C-myc, and FOXO1 in cervical cancer specimens

**Fig. 7:**
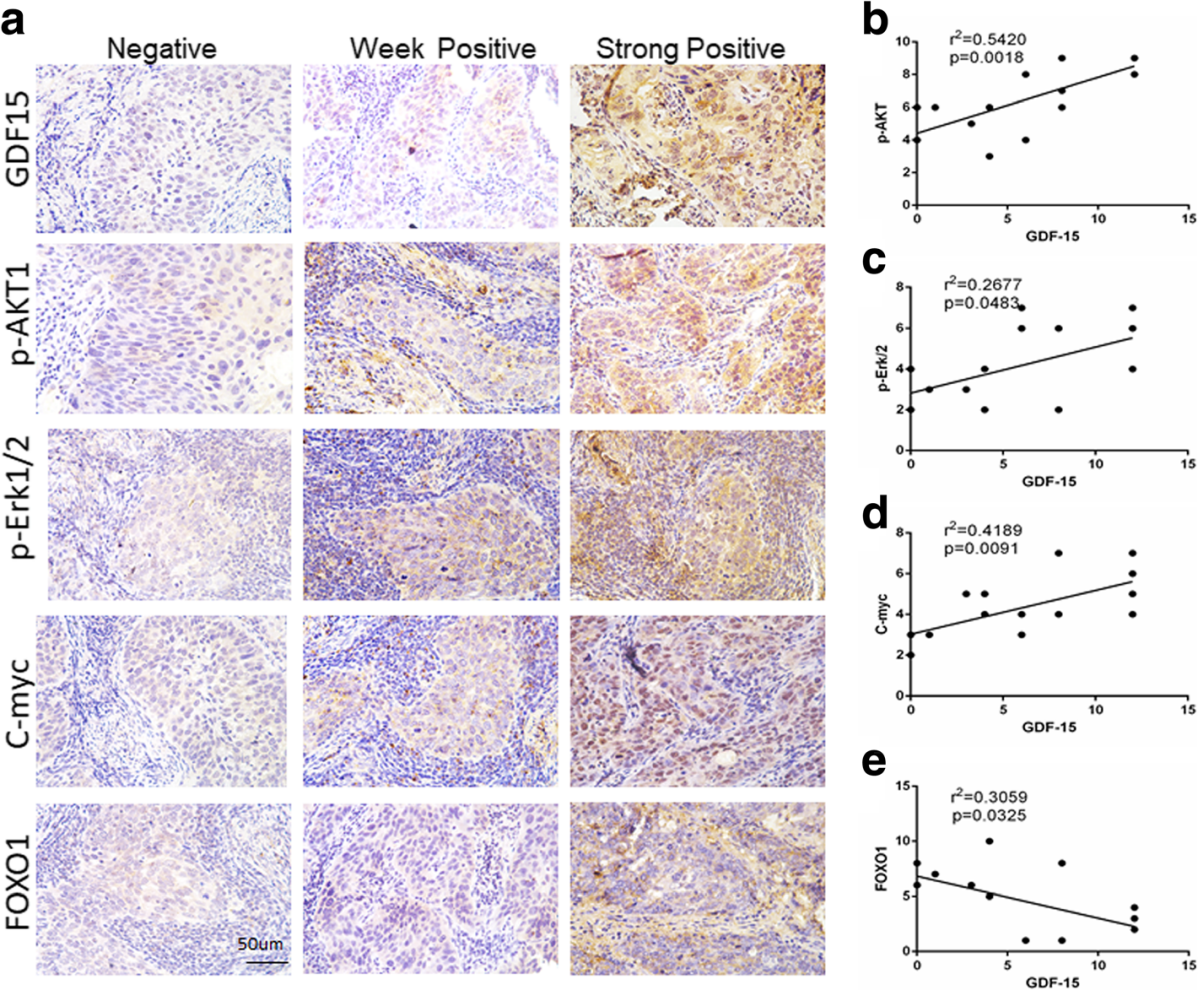
GDF15 is positively-correlated with the expression of AKT/MAPK signaling-related proteins in human cervical cancer tissues. Sixteen cervical cancer specimens were analyzed by IHC, and the representative expression of GDF15, p-AKT1, p-Erk/2, C-myc and FOXO1 is shown (**a**), scale bar, 50 μm. Correlation of GDF15 and p-AKT1, p-Erk/2, C-myc and FOXO1 expression, respectively in human cervical cancer was analyzed (**b**, **c**, **d** and **e**). Correlation analysis was performed using Pearson Chi-Square test

To validate the correlation between the expression of GDF15 and PI3K/AKT and MAPK/ERK signaling-related proteins in cervical cancer specimens, the expression of GDF15, p-AKT, p-Erk1/2, C-myc and FOXO1 was detected by IHC (Fig. 7a). Notably, a Pearson correlation analysis showed that GDF15 expression positively correlated with the expression of p-AKT, p-Erk1/2 and C-myc in 16 cervical cancer samples (r^2^ = 0.54, *P* = 0.002; r^2^ = 0.27, *P* = 0.048; r^2^ = 0.42, *P* = 0.009, respectively, Fig. 7b-d). However, the expression of GDF15 negatively correlated with the expression of FOXO1 (r^2^ = 0.31, *P* = 0.033, Fig. 7e). Therefore, these results indicated that GDF15 was associated with increased activity of the PI3K/AKT and MAPK/ERK signaling pathways in human cervical cancer.

## Discussion

GDF15 was first discovered independently by several different laboratories in the late 1990s and was found to be a regulator of macrophage activation and, a putative placental mediator of embryonic development. Recently, GDF15 was reported to have an important function in various types of carcinomas. Large-scale delineation of biomarkers from cancer samples showed elevated expression of GDF15 in the tissue and serum of patients with prostate, breast and colorectal carcinomas [24]. Consistently, GDF15 was reported to be highly expressed in prostate cancer [25], malignant melanoma [26], ovarian cancer [27] and pancreatic ductal adenocarcinoma [28]. GDF15 was thus considered a novel potential diagnostic and prognostic biomarker for the improved risk assessment of cancer progression. In the present study, GDF15 was found to be up-regulated between normal cervix and cervical cancer lesions, which suggests that GDF15 might play a role in cervical carcinogenesis. GDF15 expression was also found to be elevated in cervical cancer compared to adjacent normal tissue in a microarray-based study [12]. Although the expression of GDF15 was higher in invasive squamous cervical cancer than in high-grade squamous intraepithelial lesions in the present study, no significant difference was observed between these two groups. This lack of a difference might be attributed to the limited samples used in the present study, and a large-scale study with more specimens is needed. Therefore, all of these results imply that GDF15 is involved in cervical carcinogenesis.

The present study showed that GDF15-overexpression in HeLa and SiHa cells enhanced tumor formation in vivo. Moreover, rhGDF15, treatment with conditional medium and GDF15-overexpression stimulated HeLa and SiHa cell proliferation in vitro. Furthermore, the down-regulation of GDF15 in HT-3 cells inhibited cell proliferation in vitro. Studies have shown that GDF15 promotes cell proliferation and tumor formation in ovarian cancer, pancreatic ductal adenocarcinoma and prostate cancer [29–32]. All of these data are in accordance with each other and support the idea that GDF15 is a tumor promoter in several human cancers.

Cell cycle analysis showed that GDF15 overexpression accelerated the transition of cervical cancer cells from G0/G1 phase to S phase, whereas GDF15 down-regulation hindered the transition of cervical cancer cells from G0/G1 phase to S phase. These results suggest that GDF15 regulates the cell cycle through cell cycle checkpoint proteins. Among the cell cycle checkpoint proteins that we tested, the expression of cdc25A, CDK2, CDK4, CyclinD1 and CyclinE1 was up-regulated, and the expression of p21 was down-regulated in GDF15-overexpressing cervical cancer cells, which suggests that the cell cycle transition from G0/G1 phase to S phase in cervical cancer cells is regulated by GDF15 through the cdc25A/CDK4-CyclinD1 and p21/CDK2-CyclinE1 complexes. Consistently, Jin YJ et al. demonstrated that GDF15 promoted cell proliferation via the up-regulation of CyclinD1 and CyclinE1 and the down-regulation of p21 in human umbilical vein endothelial cells [33]. In addition, our current study showed the down-regulated expression of FOXO1 and the up-regulated expression of C-myc in GDF15-overexpressing cervical cancer cells. These data indicate that alterations in cell cycle transition and the expression of cell cycle checkpoint proteins induced by GDF15 are mediated through FOXO1 and C-myc and innovatively demonstrate the positive feedback relationship of GDF15/C-myc/GDF15 in cervical cancer cells.

Activated AKT1 [34–36] and Erk1/2 [37, 38] phosphorylate FOXO1 and C-myc and thereby promote cell survival and proliferation. In the present study, the expression of PI3K, p-AKT1, p-Erk1/2 and Ras-GTP was significantly increased by rhGDF15 treatment or GDF15 overexpression but was decreased by GDF15 knock down, which indicates that GDF15 promotes cervical cancer cell proliferation through the PI3K/AKT and MAPK/ERK signaling pathways. Additionally, rhGDF15 and GDF15 overexpression obviously increased the expression of p-ErbB2, p-AKT1 and p-Erk1/2. Furthermore, ErbB2 inhibition significantly blocked the PI3K/AKT and MAPK/ERK signaling pathways, while inhibition of the TGF-β receptor had no effects on these two pathways. Kim KK et al. have reported that GDF15 activates AKT and ERK-1/2 via the transactivation of ErbB2 in human breast and gastric cancer cells in vitro [19]. Similar results were reported by Joshi JP et al. in the breast cancer cells in vitro [30]. In our study, for the first time, an IP assay showed that GDF15 bound to the p-ErbB2 receptor. However, the current study also revealed a suppressed proliferation of HeLa and SiHa cells with TGF-βR inhibitor treatment, which suggests that TGF-βR promotes cervical cancer cell proliferation through other mechanisms. These results suggest that GDF15 activates the PI3K/AKT and MAPK/ERK signaling pathways primarily through ErbB2.

A recent study found that GFRAL was the receptor for GDF15, and this ligand promoted weight loss in mice and nonhuman primates [39–41]. We also noted that nude mice injected with GDF15-overexpressing cervical cells had less food intake and lighter body weight than mice receiving control cells, although the data are not shown in this article.

## Conclusions

**Fig. 8:**
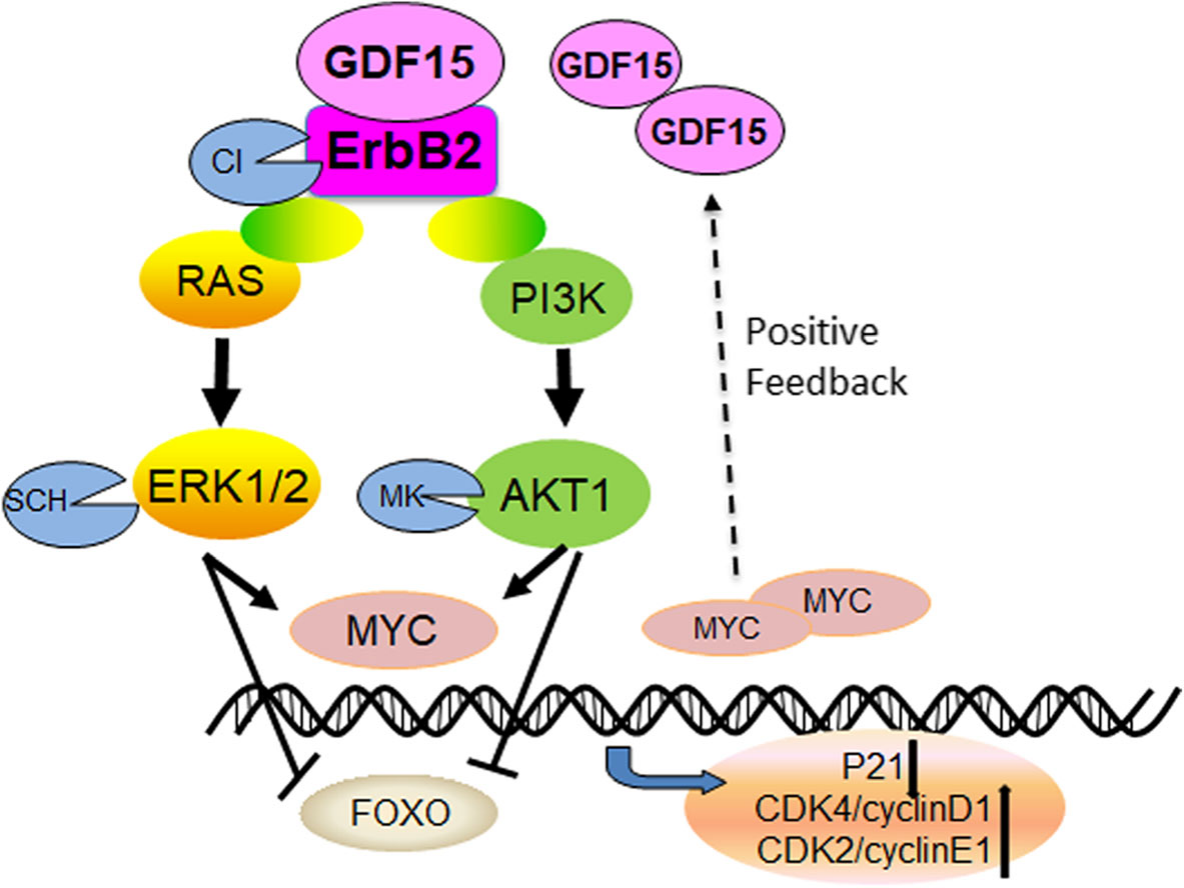
The Schematic diagram of GDF15 activating the PI3K/AKT and MAPK pathway in cervical cancer progression. GDF15 promotes cell proliferation and tumor formation in cervical cancer by which GDF15 forms a protein complex with ErbB2 and activates PI3K/AKT and MAPK pathways and reveals the mechanism about the change of cell cycle associated molecules such as p21, CDK4/CyclinD1, CDK2/CylinE1

In conclusion, this is the first study to demonstrate that GDF15 activates the PI3K/AKT and MAPK/ERK signaling pathways in a complex with ErbB2, which then alters the expression of cell cycle regulators including p21, CDK2/4 and CyclinD1/E1, and ultimately promotes cell proliferation in human cervical cancer (Fig. 8).

## Additional files

Additional file 1: **Table S1.** List of primer sequences used for vector construction in this study of Experimental Procedures. (DOC 50 kb)

Additional file 2: **Table S2.** List of primer sequences used for real time-PCR assays in this study of Experimental Procedures. (DOC 45 kb)

Additional file 3: **Figure S1.** GDF15 promotes the proliferation of human cervical cancer cell lines in vitro. GDF15 expression in human cervical cancer cell lines was detected using immunocytochemistry (a) and western blotting (b). The viability of HeLa and SiHa cells were treated with different concentration of rhGDF15 or HSA (c and d, respectively) or conditioned medium from HeLa and SiHa modified cells (e and f, respectively). HL-60 cells were used as the negative control cell line for immunostaining for GDF15. Anti-GDF15 antibody and Isotype Control antibody were used in the GDF15 and Control group respectively. HeLa-GFP and SiHa-GFP supernatant was from cells transfected with the empty pIRES2-AcGFP vector, while HeLa-GDF15 and SiHa-GDF15 supernatant was from cells transfected with the pIRES2-AcGFP-GDF15 vector. (TIFF 2140 kb)

Additional file 4: **Figure S2.** GDF15 induced activation of AKT1 and ERK1/2 in human cervical cancer cell lines. GDF15, PI3K, AKT/p-AKT1 and Erk1/2/p-Erk1/2, GAPDH were determined by western blotting and Ras-GTP was by immunoprecipitation. Western blotting of cell lysates from HeLa and SiHa cells which were treated with 0, 10, 100 ng/ml of rhGDF15 for 24 h (a and b) and the quantitative analysis were shown (c and d). Western blotting of cell lysates from HeLa-GDF15 and SiHa-GDF15 and their control cell lines (e and f) and the quantitative analysis are shown (g and h). (TIFF 1052 kb)

Additional file 5: **Figure S3.** Inhibition of ERBB2 eliminated the activation of AKT1 and ERK1/2 induced by GDF15. Western blotting of cell lysates from HeLa-GDF15, SiHa-GDF15 and their control cells which were treated with CI-1033 (a) and the quantitative analysis were shown (b and c). The data were shown as the mean ± SD of three independent experiments. *p < 0.05, **p < 0.01 vs. control using One-Way ANOVA. (TIFF 769 kb)

## Abbreviations

CDK2: Cyclin-dependent kinase-2
CDK4: Cyclin-dependent kinase-4
GDF15: Growth differentiation factor 15
MAPK: Mitogen-activated protein kinase
PI3K: Phosphoinositide 3-kinase
SCC: Squamous cell carcinoma
TGF-β: Transforming growth factor β

## References

[1] Ferlay J, Shin HR, Bray F (2010). Estimates of worldwide burden of cancer in 2008: GLOBOCAN 2008. Int J Cancer.

[2] Narisawa-Saito M, Kiyono T (2007). Basic mechanisms of high-risk human papillomavirus-induced carcinogenesis: roles of E6 and E7 proteins. Cancer Sci.

[3] Moody CA, Laimins LA (2010). Human papillomavirus oncoproteins: pathways to transformation. Nat Rev Cancer.

[4] Cao HZ, Liu XF, Yang WT (2017). LGR5 promotes cancer stem cell traits and chemoresistance in cervical cancer. Cell Death Dis.

[5] Liu XF, Li XY, Zheng PS (2018). DAX1 promotes cervical cancer cell growth and tumorigenicity through activation of Wnt/beta-catenin pathway via GSK3beta. Cell Death Dis.

[6] Yang WT, Zheng PS (2012). Kruppel-like factor 4 functions as a tumor suppressor in cervical carcinoma. Cancer.

[7] Cui N, Yang WT, Zheng PS (2016). Slug inhibits the proliferation and tumor formation of human cervical cancer cells by up-regulating the p21/p27 proteins and down-regulating the activity of the Wnt/beta-catenin signaling pathway via the trans-suppression Akt1/p-Akt1 expression. Oncotarget.

[8] Unsicker K, Spittau B, Krieglstein K (2013). The multiple facets of the TGF-beta family cytokine growth/differentiation factor-15/macrophage inhibitory cytokine-1. Cytokine Growth Factor Rev.

[9] Mimeault M, Batra SK (2010). Divergent molecular mechanisms underlying the pleiotropic functions of macrophage inhibitory cytokine-1 in cancer. J Cell Physiol.

[10] Brugge J, Hung MC, Mills GB (2007). A new mutational AKTivation in the PI3K pathway. Cancer Cell.

[11] Yang CZ, Ma J, Zhu DW (2014). GDF15 is a potential predictive biomarker for TPF induction chemotherapy and promotes tumorigenesis and progression in oral squamous cell carcinoma. Ann Oncol.

[12] Wan F, Miao X, Quraishi I (2008). Gene expression changes during HPV-mediated carcinogenesis: a comparison between an in vitro cell model and cervical cancer. Int J Cancer.

[13] Cong L, Ran FA, Cox D (2013). Multiplex genome engineering using CRISPR/Cas systems. Science.

[14] Huang H, Tindall DJ (2007). Dynamic FoxO transcription factors. J Cell Sci.

[15] Zajac-Kaye M (2001). Myc oncogene: a key component in cell cycle regulation and its implication for lung cancer. Lung Cancer.

[16] Hwang-Verslues WW, Sladek FM (2008). Nuclear receptor hepatocyte nuclear factor 4alpha1 competes with oncoprotein c-Myc for control of the p21/WAF1 promoter. Mol Endocrinol.

[17] Roy SK, Srivastava RK, Shankar S (2010). Inhibition of PI3K/AKT and MAPK/ERK pathways causes activation of FOXO transcription factor, leading to cell cycle arrest and apoptosis in pancreatic cancer. J Mol Signal.

[18] Zhao Q, Assimopoulou AN, Klauck SM (2015). Inhibition of c-MYC with involvement of ERK/JNK/MAPK and AKT pathways as a novel mechanism for shikonin and its derivatives in killing leukemia cells. Oncotarget.

[19] Kim KK, Lee JJ, Yang Y (2008). Macrophage inhibitory cytokine-1 activates AKT and ERK-1/2 via the transactivation of ErbB2 in human breast and gastric cancer cells. Carcinogenesis.

[20] Arteaga CL, Engelman JA (2014). ERBB receptors: from oncogene discovery to basic science to mechanism-based cancer therapeutics. Cancer Cell.

[21] Avraham R, Yarden Y (2011). Feedback regulation of EGFR signalling: decision making by early and delayed loops. Nat Rev Mol Cell Biol.

[22] Yarden Y, Shilo BZ (2007). SnapShot: EGFR signaling pathway. Cell.

[23] Li C, Wang Q, Wang JF (2014). Transforming growth factor-beta (TGF-beta) induces the expression of chondrogenesis-related genes through TGF-beta receptor II (TGFRII)-AKT-mTOR signaling in primary cultured mouse precartilaginous stem cells. Biochem Biophys Res Commun.

[24] Welsh JB, Sapinoso LM, Kern SG (2003). Large-scale delineation of secreted protein biomarkers overexpressed in cancer tissue and serum. Proc Natl Acad Sci U S A.

[25] Chen SJ, Karan D, Johansson SL (2007). Prostate-derived factor as a paracrine and autocrine factor for the proliferation of androgen receptor-positive human prostate cancer cells. Prostate.

[26] Boyle GM, Pedley J, Martyn AC (2009). Macrophage inhibitory cytokine-1 is overexpressed in malignant melanoma and is associated with tumorigenicity. J Invest Dermatol.

[27] Griner SE, Joshi JP, Nahta R (2013). Growth differentiation factor 15 stimulates rapamycin-sensitive ovarian cancer cell growth and invasion. Biochem Pharmacol.

[28] Wang X, Li Y, Tian H (2014). Macrophage inhibitory cytokine 1 (MIC-1/GDF15) as a novel diagnostic serum biomarker in pancreatic ductal adenocarcinoma. BMC Cancer.

[29] Mehta RS, Chong DQ, Song M (2015). Association between plasma levels of macrophage inhibitory cytokine-1 before diagnosis of colorectal cancer and mortality. Gastroenterology.

[30] Joshi JP, Brown NE, Griner SE (2011). Growth differentiation factor 15 (GDF15)-mediated HER2 phosphorylation reduces trastuzumab sensitivity of HER2-overexpressing breast cancer cells. Biochem Pharmacol.

[31] Wallin U, Glimelius B, Jirstrom K (2011). Growth differentiation factor 15: a prognostic marker for recurrence in colorectal cancer. Br J Cancer.

[32] Tsui KH, Chang YL, Feng TH (2012). Growth differentiation factor-15 upregulates interleukin-6 to promote tumorigenesis of prostate carcinoma PC-3 cells. J Mol Endocrinol.

[33] Jin YJ, Lee JH, Kim YM (2012). Macrophage inhibitory cytokine-1 stimulates proliferation of human umbilical vein endothelial cells by up-regulating cyclins D1 and E through the PI3K/Akt-, ERK-, and JNK-dependent AP-1 and E2F activation signaling pathways. Cell Signal.

[34] Boreddy SR, Pramanik KC, Srivastava SK (2011). Pancreatic tumor suppression by benzyl isothiocyanate is associated with inhibition of PI3K/AKT/FOXO pathway. Clin Cancer Res.

[35] Yamagata K, Daitoku H, Takahashi Y (2008). Arginine methylation of FOXO transcription factors inhibits their phosphorylation by Akt. Mol Cell.

[36] Xin B, Yamamoto M, Fujii K, et al. Critical role of Myc activation in mouse hepatocarcinogenesis induced by the activation of AKT and RAS pathways. Oncogene. 2017;36:5087-97 10.1038/onc.2017.114 PMC5596209 28481866

[37] Pan CC, Bloodworth JC, Mythreye K (2012). Endoglin inhibits ERK-induced c-Myc and cyclin D1 expression to impede endothelial cell proliferation. Biochem Biophys Res Commun.

[38] Shankar S, Chen Q, Srivastava RK (2008). Inhibition of PI3K/AKT and MEK/ERK pathways act synergistically to enhance antiangiogenic effects of EGCG through activation of FOXO transcription factor. J Mol Signal.

[39] Yang L, Chang CC, Sun Z (2017). GFRAL is the receptor for GDF15 and is required for the anti-obesity effects of the ligand. Nat Med.

[40] Emmerson PJ, Wang F, Du Y (2017). The metabolic effects of GDF15 are mediated by the orphan receptor GFRAL. Nat Med.

[41] Mullican SE, Lin-Schmidt X, Chin CN (2017). GFRAL is the receptor for GDF15 and the ligand promotes weight loss in mice and nonhuman primates. Nat Med.

