# Supplementary material for: GDF15 promotes the proliferation of cervical cancer cells by phosphorylating AKT1 and Erk1/2 through the receptor ErbB2
Source: J Exp Clin Cancer Res. 2018 Apr 10;37:80. doi: 10.1186/s13046-018-0744-0 (PMC5894198; doi:10.1186/s13046-018-0744-0)
Supplement: Supplementary file 1 — Table S1. List of primer sequences used for vector construction in this study of Experimental Procedures. (DOC 50 kb) [file 13046_2018_744_MOESM1_ESM.doc]

**Supplementary Table S1: List of primer sequences used for vector construction** **in this study of Experimental Procedures.**

**GDF15 CDS full long PCR, GDF15-specific and ErbB2-specific short hairpin RNA (shRNA) and sgRNA of CRISPR/CAS9 system.**

| Name | Sequences |
| --- | --- |
| GDF15-CDS-S | GGAAGATCTGCCACCATGCCCGGGCAAGAAC |
| GDF15-CDS-A | CCGGAATTCTCATATGCAGTGGCAGTCTTTG |
| shGDF15-41-S | CACCGCAAGAACTCAGGACGGTGAATTCAAGAGATTCACCGTCCTGAGTTCTTGCTTTTTTG |
| shGDF15-41-A | GATCCAAAAAAAGCAAGAACTCAGGACGGTGAATCTCTTGAATTCACCGTCCTGAGTTCTTGC |
| shGDF15-531-S | CACCGACCAACTGCTGGCAGAATCTTTCAAGAGAAGATTCTGCCAGCAGTTGGTCTTTTTTG |
| shGDF15-531-A | GATCCAAAAAAGACCAACTGCTGGCAGAATCTTCTCTTGAAAGATTCTGCCAGCAGTTGGTC |
| shErbB2-804-S | CACCGCTCTTTGAGGACAACTATGCTTCAAGAGAGCATAGTTGTCCTCAAAGAGCTTTTTTG |
| shErbB2-804-A | GATCCAAAAAAGCTCTTTGAGGACAACTATGCTCTCTTGAAGCATAGTTGTCCTCAAAGAGC |
| shErbB2-1020-S | CACCGCTGGCTCTCACACTGATAGATTCAAGAGATCTATCAGTGTGAGAGCCAGCTTTTTTG |
| shErbB2-1020-S | GATCCAAAAAAGCTGGCTCTCACACTGATAGATCTCTTGAATCTATCAGTGTGAGAGCCAGC |
| shNC-A | CACCGTTCTCCGAACGTGTCACGTTTCAAGAGAACGTGACACGTTCGGAGAATTTTTTG |
| shNC-S | GATCCAAAAAATTCTCCGAACGTCTCACGTTCTCTTGAAACGTGACACGTTCGGAGAAC |
| | GDF-15-1T | | --- | | CACCGGAGTTGCGGAAACGCTACG |
| | GDF-15-1B | | --- | | AAACCGTAGCGTTTCCGCAACTCC |
| | GDF-15-2T | | --- | | CACCGAAACTTGCGCGGCTCGCCT |
| | GDF-15-2B | | --- | | AAACAGGCGAGCCGCGCAAGTTTC |
| | GDF-15-3T | | --- | | AAACAGGCGAGCCGCGCAAGTTTC |
| | GDF-15-3B | | --- | | AAACAGAGTTGCGGAAACGCTACC |
| GDF-15-4T | CACCGTTCCGCAACTCTCGGAATC |
| GDF-15-4B | AAACGATTCCGAGAGTTGCGGAC |
