# Supplementary material for: GDF15 promotes the proliferation of cervical cancer cells by phosphorylating AKT1 and Erk1/2 through the receptor ErbB2
Source: J Exp Clin Cancer Res. 2018 Apr 10;37:80. doi: 10.1186/s13046-018-0744-0 (PMC5894198; doi:10.1186/s13046-018-0744-0)
Supplement: Supplementary file 2 — Table S2. List of primer sequences used for real time-PCR assays in this study of Experimental Procedures. (DOC 45 kb) [file 13046_2018_744_MOESM2_ESM.doc]

**Supplementary Table S2: List of primer sequences used for real time-PCR assays in this study of Experimental Procedures**

| Name | Sequences |
| --- | --- |
| CDC2-F | TGATCCAGCCAAACGAATTTC |
| CDC2-R | GCTACATCTTCTTAATCTGATTGTCCAA |
| CDC25AF | GGCAAGCGTGTCATTGTTGTG |
| CDC25AR | ACAGCTCAGGGTAGTGGAGTTTGG |
| CDK2-F | GCTAGCAGACTTTGGACTAGCCAG |
| CDK2-R | AGCTCGGTACCACAGGGTCA |
| CDK4-F | ATGTTGTCCGGCTGATGGA |
| CDK4-R | CACCAGGGTTACCTTGATCTCC |
| CyclinA1-F | GTTGTGCTGGCTACAGTGG |
| CyclinA1-R | CCTGCTCTAGTTCATCCATGTA |
| CyclinB1-F | TCTGGATAATGGTGAATGGACA |
| CyclinB1-R | CGATGTGGCATACTTGTTCTTG |
| CyclinD1-F | AAACAGATCATCCGCAAACAC |
| CyclinD1-R | GTTGGGGCTCCTCAGGTTC |
| P16-F | CATAGATGCCGCGGAAGGT |
| P16-R | CCCGAGGTTTCTCAGAGCCT |
| P21-F | GCAGACCAGCATGACAGATTTC |
| P21-R | CGGATTAGGGCTTCCTCTTG |
| P53-F | AAGAAACTGGCGGAATGGC |
| P53-R  GDF15-P1-F  GDF15-P1-R  GDF15-P2-F  GDF14-P2-R  GDF15-3’UTR-F  GDF15-3’UTR-R | CCAAGAACCACCCCTGAGTC  GTAATCCCAGCTACTAGGGAGGATG  CACAGCCCTGTCCTAGGGGAT  TTAGTAGAGACAGGGTTTCTCCATG  CATCTCTAAAAAAAAGAATTCTGCG  AAACAGCTGAGACCCCATACCT  CCAAAGAGAGAAGTTTTGAGGAATG |
