# Supplementary figures and images for: GDF15 promotes the proliferation of cervical cancer cells by phosphorylating AKT1 and Erk1/2 through the receptor ErbB2
Source: J Exp Clin Cancer Res. 2018 Apr 10;37:80. doi: 10.1186/s13046-018-0744-0 (PMC5894198; doi:10.1186/s13046-018-0744-0)

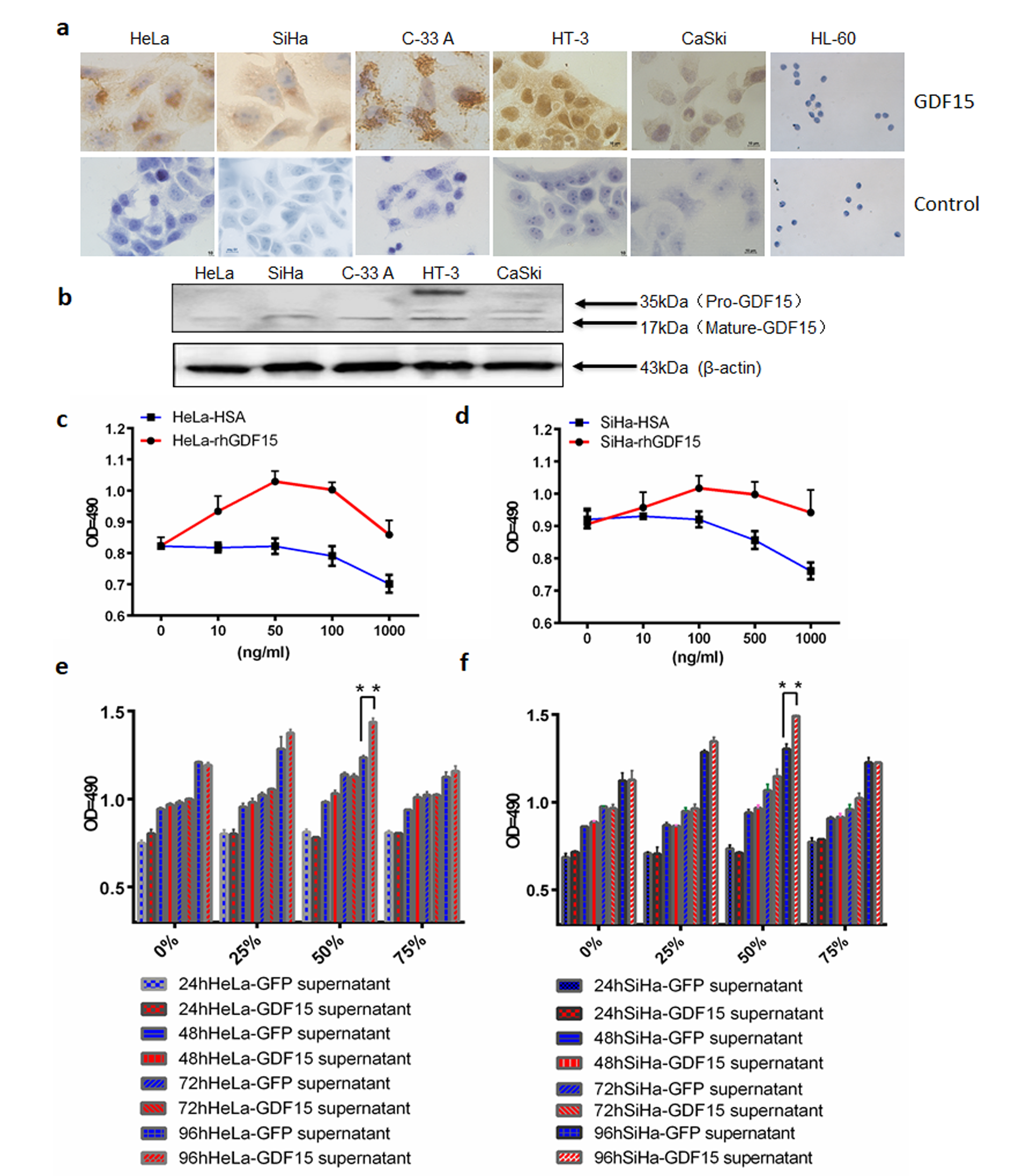

Supplement: Supplementary file 3 — Figure S1. GDF15 promotes the proliferation of human cervical cancer cell lines in vitro. GDF15 expression in human cervical cancer cell lines was detected using immunocytochemistry (a) and western blotting (b). The viability of HeLa and SiHa cells were treated with different concentration of rhGDF15 or HSA (c and d, respectively) or conditioned medium from HeLa and SiHa modified cells (e and f, respectively). HL-60 cells were used as the negative control cell line for immunostaining for GDF15. Anti-GDF15 antibody and Isotype Control antibody were used in the GDF15 and Control group respectively. HeLa-GFP and SiHa-GFP supernatant was from cells transfected with the empty pIRES2-AcGFP vector, while HeLa-GDF15 and SiHa-GDF15 supernatant was from cells transfected with the pIRES2-AcGFP-GDF15 vector. (TIFF 2140 kb) [file 13046_2018_744_MOESM3_ESM.tif]

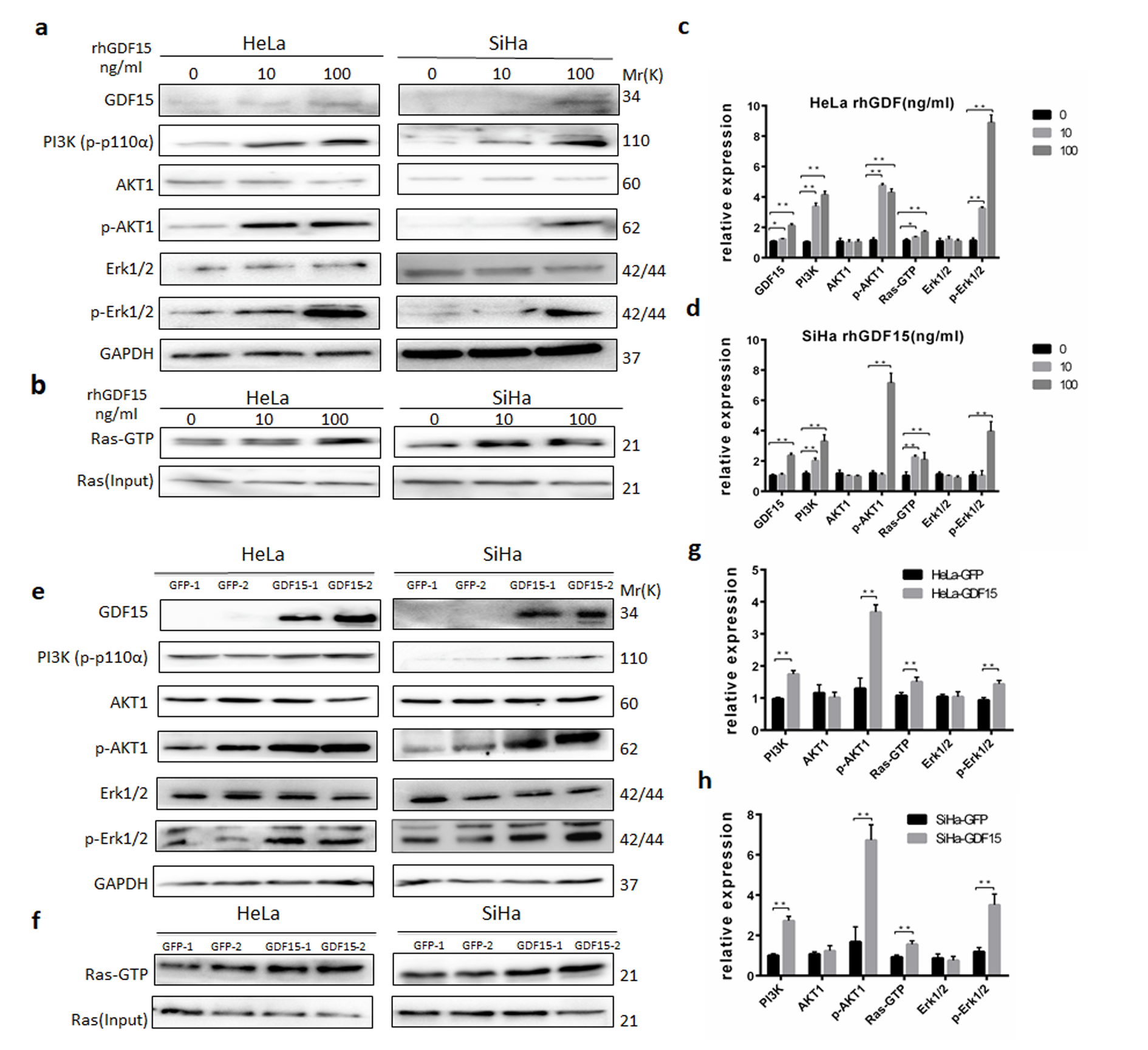

Supplement: Supplementary file 4 — Figure S2. GDF15 induced activation of AKT1 and ERK1/2 in human cervical cancer cell lines. GDF15, PI3K, AKT/p-AKT1 and Erk1/2/p-Erk1/2, GAPDH were determined by western blotting and Ras-GTP was by immunoprecipitation. Western blotting of cell lysates from HeLa and SiHa cells which were treated with 0, 10, 100 ng/ml of rhGDF15 for 24 h (a and b) and the quantitative analysis were shown (c and d). Western blotting of cell lysates from HeLa-GDF15 and SiHa-GDF15 and their control cell lines (e and f) and the quantitative analysis are shown (g and h). (TIFF 1052 kb) [file 13046_2018_744_MOESM4_ESM.tif]

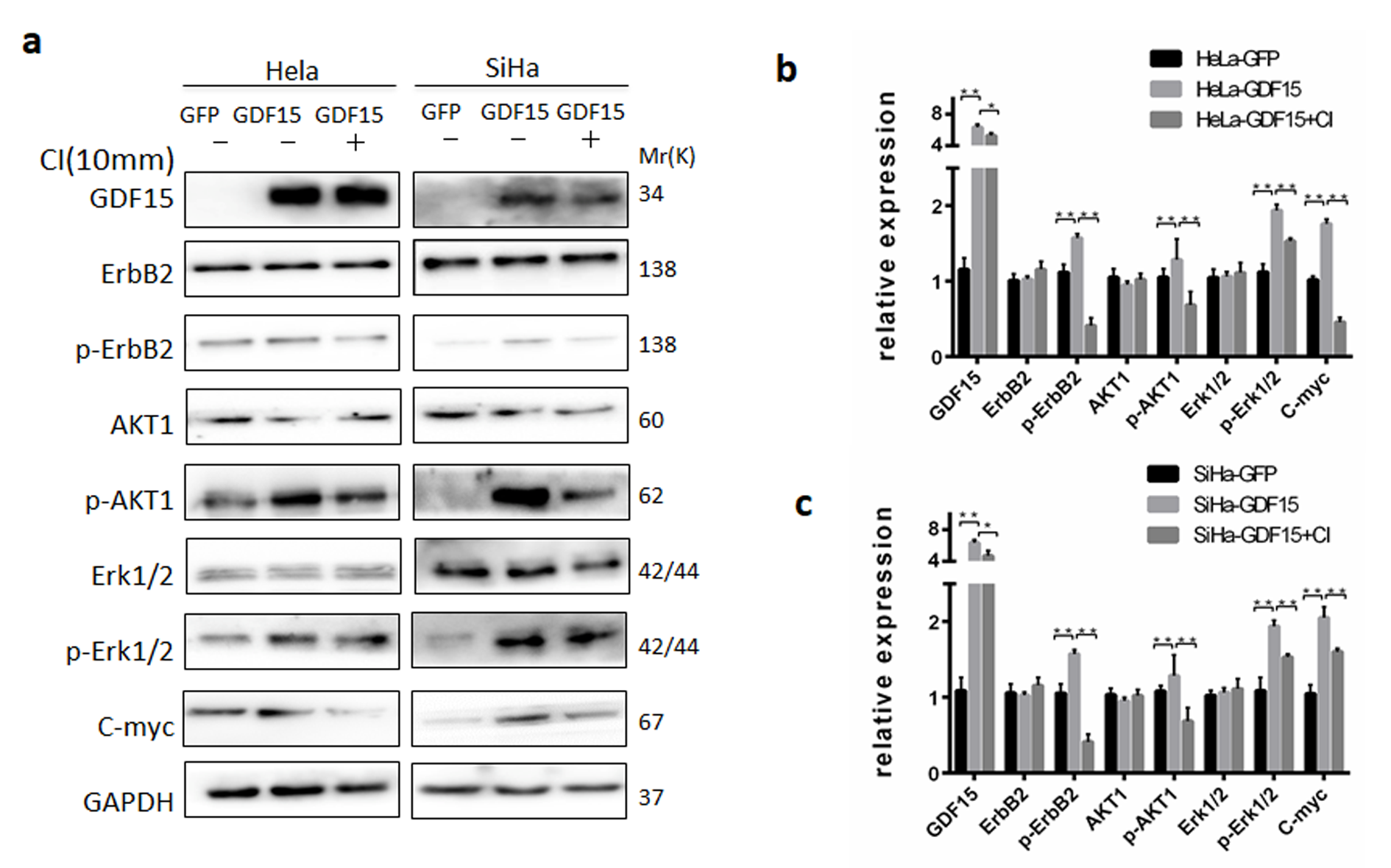

Supplement: Supplementary file 5 — Figure S3. Inhibition of ERBB2 eliminated the activation of AKT1 and ERK1/2 induced by GDF15. Western blotting of cell lysates from HeLa-GDF15, SiHa-GDF15 and their control cells which were treated with CI-1033 (a) and the quantitative analysis were shown (b and c). The data were shown as the mean ± SD of three independent experiments. *p < 0.05, **p < 0.01 vs. control using One-Way ANOVA. (TIFF 769 kb) [file 13046_2018_744_MOESM5_ESM.tif]
